# 30–40 mmHg of Compression

**DOI:** 10.1007/s11606-026-10451-1

**Published:** 2026-04-21

**Authors:** Andrea McGowan

**Affiliations:** University of Michigan Medical School, Ann Arbor, MI USA

When I started medical school, I vigorously shook the dust off of my academic prowess. I had changed career paths from public health to medicine and hadn’t studied the basic sciences for several years. I left my competitive academic edge back in high school, where I sacrificed my first kiss at Prom for five AP courses and dozens of leadership positions. My approach to schooling as a young adult was much more balanced with my rescue dog (Zoey) and an addiction to baking that occupied my time.

As I reentered a formal academic environment and learned how to study again, I marveled at my peers’ intelligence and work ethic. It seemed like they could memorize every droplet of the figurative curricular firehose while publishing three first author papers and founding a non-profit. I, on the other hand, felt like I was drowning in an ocean of curricular content. I didn’t understand how a person with so many shortcomings could exist in a class full of model students.

During the preclinical year at my school, faculty members occasionally shared bits and pieces of their own imperfections that I scooped up and savored like a starving street rat. One prestigious neurologist shared his experience as a medical student granting the request of a woman in labor who felt the urge to have a bowel movement, only to discover that he was about to deliver her baby into a bedpan. Another shared scathing feedback she received about not being cut out for a procedural field, now leading her department as a renowned surgeon.

It’s harder for students to disclose failures like this. There is no legacy built on their successful careers as clinicians with 125 first author publications and multiple promotions. Their legacy is only as deep as the three block exams they managed to pass and whatever extra-curricular they had on their resume from college.

On week 3 of my clinical year, perfection was already out of reach. I had fumbled my way through the first 3 weeks of my psychiatry rotation. One aspect of our curriculum was to shadow electroconvulsive therapy. After arriving to the hospital without a sense of what to expect, I stepped into the procedure room and met the anesthesiologist. He shared some information about the procedure and the basics of bag masking. The patient received one convulsive shock and the next thing I knew, my vision started fading. My legs wobbled, heart raced, and palms sweated.

Somehow, in my pre-syncopal confusion, I had stepped out of the procedure room. In a panic, I was looking for a surface to lean up against as my vision cut out. However, this natural instinct resulted in me essentially running full speed into a wall like a cartoon character. I awakened, slumped on the ground while throwing up and bleeding profusely out of my nose. As I got wheeled off to the emergency department, I waved to the attending and said, “I’ll be back!” He chuckled. My stomach sank out of embarrassment. I got discharged from the emergency department with stitches in my nose, IV fluids, and a day off from the rotation.

Not only was I embarrassed, but I was defeated. This failure brought back that sensation of drowning, telling me that I was incapable of making it through clinical year. I passed out during my psychiatry rotation, hardly 1 month into the foundation of my clinical education. No open abdominal surgery or horrible smell. Simply a patient with treatment-resistant depression. What I didn’t realize was that this blunder created an opportunity. I couldn’t hide my vulnerability. It was quite literally written on my face with seven stitches, a band-aid, and bags under my eyes the next day in lecture. When classmates approached with concern and curiosity, I met them with honesty and humor disclosing my vasovagal episode. Sharing this embarrassment started an unexpected dialogue with my previously perfect classmates. Many of them were also having their own failures during this transition into the clinical world. From failing to meet expectations to following their resident into the bathroom, we understood each other. We just needed permission to share these mistakes.

As I approach the end of medical school, I think about this moment often and the power I unlocked by embracing my embarrassment, simply because I couldn’t hide it. Vulnerability helped me connect with my peers and begin to dismantle the façade of perfectionism. Now, I look for opportunities to normalize failures with younger students and peers. My humanity is no longer a shame but the strength that keeps me standing. That and the 30–40 mmHg compression stockings that I pull up to my knees each day.

